# Challenges on the development of a dengue vaccine: a comprehensive review of the state of the art

**DOI:** 10.1099/jgv.0.001831

**Published:** 2023-03-01

**Authors:** Jessica Pintado Silva, Ana Fernandez-Sesma

**Affiliations:** ^1^​ Department of Microbiology, Icahn School of Medicine at Mount Sinai, 1 Gustave Levy Place, New York, NY 10029, USA; ^2^​ Graduate School of Biomedical Sciences, Icahn School of Medicine at Mount Sinai, 1 Gustave Levy Place, New York, NY 10029, USA

**Keywords:** Dengue, immunology, vaccines

## Abstract

Dengue virus (DENV) is the mosquito-borne virus of greatest human health concern. There are four serotypes of DENV (1-4) that co-circulate in endemic areas. Each serotype of DENV is individually capable of causing the full spectrum of disease, ranging from self-resolving dengue fever to the more severe dengue haemorrhagic fever (DHF) or dengue shock syndrome (DSS). Based on data published by the CDC, one in four people who become infected with dengue will become ill. Of those that do develop symptomology, the symptoms can range from mild to severe. Symptoms can vary from rash, ocular aches and pains to more intense symptoms in the manifestation of severe dengue. Roughly, 1 in 20 people who become ill will develop severe dengue, which can result in shock, internal bleeding and death. There is currently no specific treatment for dengue and only one licensed vaccine (Dengvaxia) for children 9 through 16 years of age in just a few countries. Despite its licensure for clinical use, Dengvaxia has performed with low efficacy in children and dengue naïve individuals and critically has resulted in increased risk of developing severe dengue in young, vaccinated recipients. Currently, there are various novel strategies for the development of a dengue vaccine. In this review we have conducted a detailed overview of the DENV vaccine landscape, focusing on nine vaccines in the pipeline to provide a comprehensive overview of the most state-of-the-art developments in strategies for vaccines against DENV.

## Abbreviations

ADE, antibody dependent enhancement; AG129, interferon alpha and gamma receptor knocked out mice; AS03_B_, 3-O-desacylcinomonophosphoryl lipid B; AS01_E_, 3-O-desacylcinomonophosphoryl lipid A; AST, aspartate transferase; ASTMH, American Society for Tropical Medicine and Hygene; ASV, American Society of Virology; CMI, cell mediated immunity; DC, dendritic cell; Dengvaxia®, Sanofi Pasteur-developed tetravalent dengue vaccine; DENV, dengue virus; DHF, dengue hemorrhagic fever; DHIPC, dengue human immunology project consortium; DMID, division of microbiology and infectious diseases; DoD, Department of Defense; DPIV, Walter Reed Army Institute of Research-developed tetravalent dengue purified inactivated vaccine; DSS, dengue shock syndrome; DSV4, tetravalent virus-like particle dengue vaccine; E, envelope protein; EDIII, E protein domain III; GSK, GlaxoSmithKline; HEK, human embryonic kidney; HIPC, human immunology project consortium; IAV, influenza A virus; IgE, immunoglobulin E; IL-, interleukin-; ISMMS, at Icahn School of Medicine at Mount Sinai; JEV, Japanese encephalitis virus; LATV, live attenuated tetravalent vaccine; LNP, lipid nanoparticle; MMR, measles, mumps, rubella vaccine; mRNA, messenger ribonucleic acid; nAb, neutralizing antibody; NGC, New Guinea C; NHP, non-human primates; NIAID, The National Institute of Allergy and Infectious Diseases; NIH, National Institutes of health; NS, non structural; PCR, poly chain reaction; PDK, primary dog kidney; PEG, poly ethylene glycol; PIV, purified inactivated virus; PLoS, public library of sciences; PrM, pre matrix protein; PRNT, Plaque Reduction Neutralization Test; RT, reverse transcriptase; TDEN, GlaxoSmithKline developed a live attenuated, tetravalent dengue vaccine; TDV, Takeda Pharmaceuticlas-produced live-attenuated tetravalnt dengue vaccine; TLAV, Walter Reed Army Institute of Research-developed tetravalent live attenuated dengue vaccine boost; TV003/TV005, The National Institute of Allergy and Infectious Diseases-developed live attenuated tetravalent dengue vaccine formulations; UTR, untranslated region; VCD, virologically confirmed dengue; VIDD, The Fred Hutch Institute Vaccine and Infectious Disease Division; WHO, World Health Organization; WRAIR, The Walter Reed Army Institute of Research; YFV, yellow fever vaccine; ZIKV, zika virus; Δ, deletion.

## Impact Statement

The incidences of dengue virus infections have grown exponentially over the last decade culminating in a significant global public health concern with the greatest burden lying in tropical and subtropical countries. Dengue disease can manifest in a range of clinical symptoms with the most severe pathogenesis resulting in severe illness and death. Currently there are no effective antiviral therapies and only one licensed vaccine in certain countries. Our review identifies current developments in the dengue vaccine pipelines as well as exploring challenges in dengue vaccine development.

## Introduction

With nearly half of the world’s population at risk of infection, dengue virus (DENV) is the arthropod borne virus of greatest human significance. Dengue virus is transmitted via the bite of an infected *Aedes Aegypti* or *Aedes Albopictus* mosquito. At the present time, there are no globally licensed antiviral treatments or vaccines that protect against all four of the DENV serotypes (DENV 1–4) [1]. DENV epidemiology is multifaceted and presents with a broad spectrum of clinical manifestations, which can range from asymptomatic disease to the life-threatening DHF and DSS [2]. Each of the four DENV serotypes is capable of causing the full spectrum of disease. Also, secondary infections with a heterologous DENV serotype can result in increased infection and enhanced disease via a mechanism known as antibody-dependent enhancement (ADE) [3, 4]. Infection with one DENV serotype confers long lasting antibodies (humoral) and T-cell responses (cellular immunity) to that serotype and provides protection. In ADE, subsequent exposure to a different DENV serotype results in the binding of pre-existing DENV antibodies to the new serotype, forming a virus-immune complex that can facilitate entry into immune cells via Fcγ receptors resulting in enhanced viral replication and viremia [3]. Because of the phenomenon of ADE, each DENV serotype must be individually considered when developing a vaccine or therapeutic. Dengvaxia, has been licensed in Mexico, the Philippines, Indonesia, Brazil, El Salvador, Costa Rica, Paraguay, Guatemala, Peru, Thailand and Singapore and in 2019 it was licensed by the FDA and approved for use by the CDC ACIP under the measure that all vaccinees had to be shown to be seropositive for dengue prior to administration of the vaccine [5]. Despite its commercialization, Dengvaxia resulted in severe adverse side effects and low effectiveness [6]. Takeda Biologics TAK-003 vaccine has been granted priority review by the U.S. Food and Drug Administration for the prevention of dengue disease caused by all four DENV serotypes in individuals 4 to 60 years of age and will be licensed in Indonesia in 2023 [7, 8]. As a result, a globally licensed and effective vaccine against DENV is still greatly needed.

## Dengue vaccine development

There have been multiple attempts at generating a vaccine against DENV, which include live-attenuated chimeric recombinant virus, live-attenuated virus, inactivated virus, recombinant protein and mRNA vaccines. Each vaccine prototype varies in its attenuation properties, efficacy and immunogenicity profile. The characteristics, efficacies and limitations of each dengue vaccine will be discussed thoroughly in this review.

## Dengvaxia

**Fig. 1.**
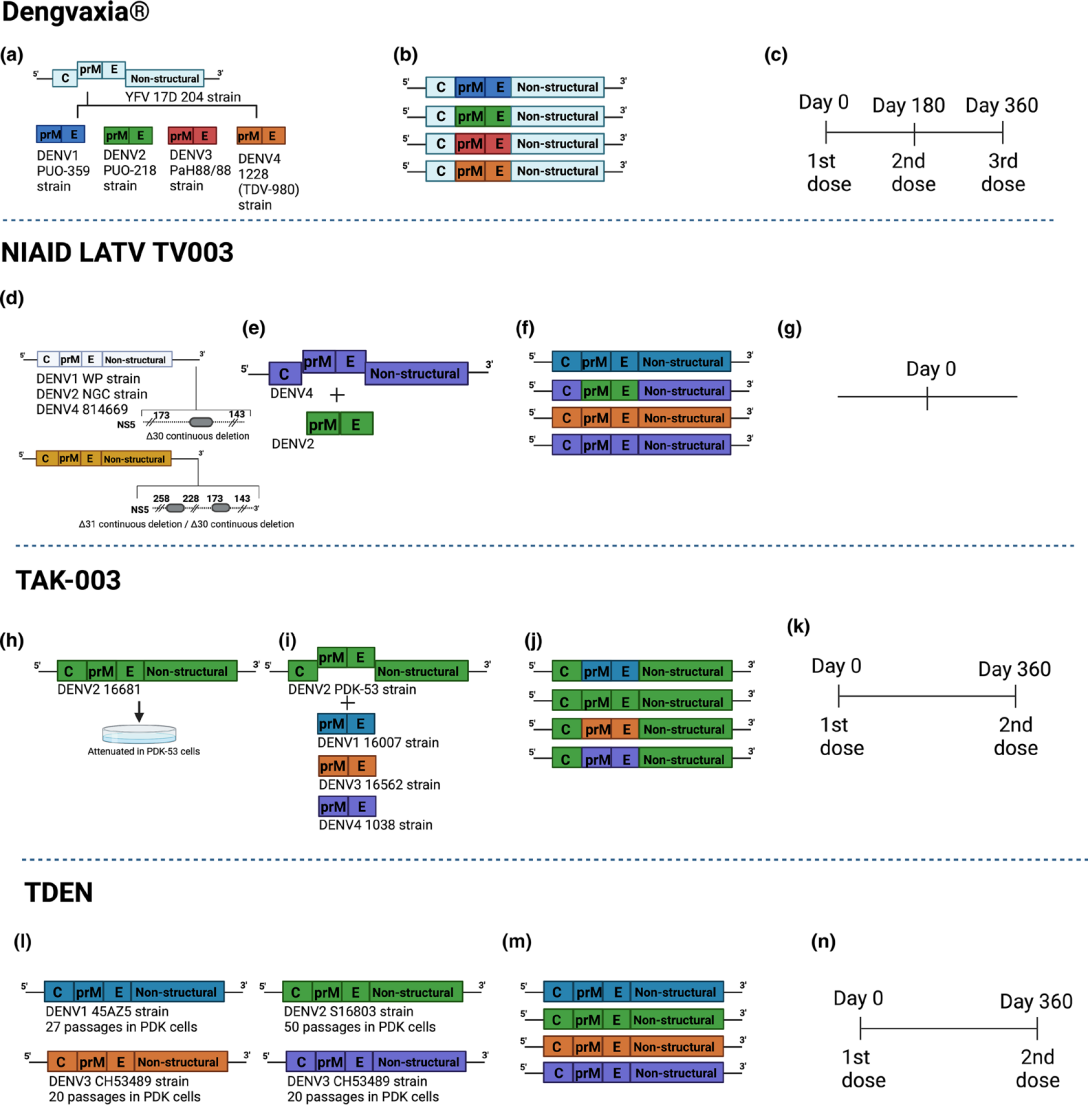
Development of live-attenuated vaccines by Sanofi, NIAID/Butantan, Takeda and U.S. Army Medical research Materiel Command. (**a**) Development of the Sanofi vaccine. Figure modified from [76]. The YFV 17D vaccine virus was used as the backbone for the generation of the chimeric viruses. The YFV 17D envelope proteins were replaced with those from wild-type DENV serotypes. (**b**) Representation of genetic construction of the four DENV vaccine viruses that compose Dengvaxia. (**c**) Dengvaxia immunization regimen based on a three-dose schedule. (**d**) Development of NIAID/Butantan vaccine. Live-attenuated vaccine viruses were generated by the introduction of 30 nucleotide deletions in the 3’ UTR of DENV1 WP, DENV2 NGC and DENV4 814 669. For the DENV3 Sleman-78 strain an additional 31 nucleotide deletion was introduced in the 3’ UTR. (**e**) The DENV2 component of LATV TV003/TV005 was generated by chimerization in which the prM and E genes from DENV2 NGC were introduced into the backbone of DENV4Δ30. (**f**) Representation of the four DENV vaccine constructs, which compose the NIAID/Butantan LATV TV003 and TV005 vaccines. (**g**) Vaccine regimen for NIAID/Butantan vaccine. (**h**) Development of Takeda’s live-attenuated recombinant vaccine. (**i**) Takeda DENV1, DENV3 and DENv4 vaccine strains were generated by introducing the prM and E proteins of each of the DENV serotypes into the DENV2 PDK53 vaccine virus. (**j**) Representation of the genetic composition of the vaccine viruses in the Takeda vaccine. (**k**) Takeda’s immunization schedule. (**l**) Development of TDEN vaccine formulation in which the four DENV strains were serially passaged in PDK cells. (**m**) Representation of genetic composition of vaccine viruses in the TDEN vaccine. Diagrams were designed using Biorender.

Sanofi Pasteur’s Dengvaxia is the first and only commercially licensed vaccine for dengue, currently being recommended and administered in 20 countries. The vaccine is a tetravalent formulation and is composed of the Sanofi Pasteur yellow fever vaccine (YFV 17D) backbone with the PrM and E proteins of DENV replacing the PrM and E proteins of YFV 17D (Fig. 1a, b) [9, 10]. As the pioneering vaccine against DENV, the Dengvaxia clinical trial and field results have been closely monitored, with special focus on the possibility of ADE. In a phase-III clinical trial, children under the age of 9 were vaccinated with a three-dose regimen of Dengvaxia with doses administered at intervals of 0, 6 and 12 months (Fig. 1c) . The efficacy of the vaccination was monitored by detection of symptomatic dengue infection at 25 months post-their 12 month vaccination. Dengvaxia showed efficacies of 65.6 % in children older than 9 years of age and 44.6 % in children younger than 9 years of age [11]. Dengvaxia completed phase-III efficacy trials with mixed results. In terms of hospitalizations, children 9 years of age or older were hospitalized at an 80.75 % rate while children 9 years of age or younger were hospitalized at a rate of 55.9 % [12]. This data suggests that Dengvaxia elicits poor protection efficacy in children under 9 years of age. In addition, Dengvaxia differed in its ability to confer protection across the four DENV serotypes in terms of virologically confirmed symptomatic dengue. Dengvaxia elicited a 50.3 % efficacy against DENV1, 42.3 % against DENV2, 74.0 % against DENV3 and 77.4 % against DENV4 [13].

Vaccine efficacy varied by DENV serotype, age of recipient, serostatus and time of vaccination. Importantly, the efficacy against DENV2 did not meet statistical significance in the CYD23 clinical trial (9.2 %; 95 % CI −75 to 51.3) or in the CYD14 trial (35 %; 95 % CI: −9.2 to 61) [14]. Data also demonstrated significantly lower efficacy in vaccine recipients who were DENV seronegative at the time of vaccination (Table 1).

**Table 1.** Dengue vaccines currently licensed or under development.

Name^a^	Year^b^	Valence^c^	Vaccine formulation	Developer/manufacturer	Evaluation	Adjuvanted
Dengvaxia	2015	Tetravalent	Chimeric viruses YFV/DEN 1–4	Sanofi Pasteur	Licensed	No
TV003/TV005	2003	Tetravalent	Three genetically attenuated viruses and one chimeric virus	NIAID^d^ and Butantan^e^	*In vivo* (phase IIIB)	No
TAK-003	2006	Tetravalent	Chimeric viruses DEN-2 PDK-53, DEN -1,–3, or −4	Takeda	*In vivo* (phase II) To be licensed in Indonesia in 2023	No
TDEN	2017	Tetravalent	Viruses attenuated with passages in PDK cells	WRAIR^f^ and GlaxosmithKline	*In vivo* (phase I-II)	No
DPIV	2012	Tetravalent	Purified inactivated viruses (DEN 1–4), Aluminium hydroxide AS01, AS03 or AS04 adjuvants	WRAIR, GllaxosmithKline and FIOcruz^g^	*In vivo* (phase I)	Yes
TVDV	2018	Tetravalent	DNA vaccine based on prM and E protein coding sequences cloned in VR1012 plasmid and co-administered with VAXFECTIN as an adjuvant	U.S. AMRDC^h^, WRAIR, NMRC and Vical	*In vivo* (animal and phase I)	Yes
V180	2018	Tetravalent	Recombinant proteins based on prM and 80 % of E protein of DEN 1–4 combined with different adjuvants	Merck and Co.	*In vivo* (phase I)	Yes
DSV4	2018	Tetravalent	Virus like particles expressing EDIII of DEN 1–4	International Centre for Genetic Engineering and Biotechnology	*In vivo* (animal)	No
E80-mRNA	2020	Tetravalent	mRNA expressing human IgE and E80 protein packaged into LNP	CAS laboratory of Molecular Virology and Immunology, Institute Pasteur of Shanghai	*In vivo* (animal)	No

^a^Current name of the vaccine formulation; ^b^Name of the vaccine formulation in its most current stage; ^c^Vaccine formulations were evaluated *in vitro* and *in vivo*. *In vivo* assays involved pre-clinical tests in animal models and or phase I, II and III clinical trials; ^d^National Institute of Allergy and Infectious Diseases, National Institutes of Health; ^e^Butantan Institute, Sāo Paulo, Brazil, ^f^Walter Reed Army Institute of Research; ^g^Oswaldo Cruz Foundation; ^h^U.S. Army Medical Research and Development Command

The results from the first year of the long-term safety study for the Dengvaxia trials demonstrated that the efficacy of Dengvaxia against hospitalization was promising with a major caveat that outcomes were highly dependent on the serostatus of DENV exposure as well as age at the time of vaccination. Further research needs to be done to understand why the efficacy of Dengvaxia is reduced in subjects who are seronegative at the time of vaccination and why the risk of hospitalization increases in children less than 9 years of age. To date, these questions remain unclear and are the topic of further investigation.

Overall, the vaccine elicited lower protection against DENV1 and DENV2 and generated an antibody response that was predominantly against DENV4 [15] . Thus Dengvaxia functionally works as a monovalent vaccine against DENV4 only. Data suggests that the infectivity and resulting immunogenicity induced from Dengvaxia may not be balanced. This imbalance is suggested by the need to give three doses of the vaccine over a 12-month period. The majority of vaccines administered parenterally including YFV and Japanese encephalitis virus (JEV) and multivalent vaccines such as measles, mumps, rubella (MMR) are delivered as a single primary dose. Revaccination may be conducted at a later time for vaccinees who did not respond to the first dose. The primary reason for the use of a single vaccine dose is that the primary dose introduces replicating vaccine virus that elicits neutralizing antibodies that are capable of blocking infection and replication of a subsequent live virus dose [16]. Interestingly, if a second dose does elicit a significant boost in antibody titre it is likely due to a sub-par primary response that was insufficient and unable to neutralize vaccine virus and subsequently allows for replication following the second dose [17]. Ultimately, a weak primary dose would be incapable of protecting against infection when challenged with a second dose of live vaccine virus. The frequencies of seroconversion to the four DENV serotypes varied greatly; seroconversion to DENV-1 was 33%, DENV-48%, DENV3 were 80 % and DENV4 was 85 % [18]. A subset of vaccinees in the study were further examined to determine the levels of viremia post-vaccination. Viremia was detected at day 7 in 57.7 % of vaccine recipients of which 86.7 % was due to the DENV4 component of Dengvaxia [14]. Only 1/25 vaccinees had the DENV2 component of Dengvaxia recovered after a single dose [11]. Notably, the DENV1 component was not recovered in any of the vaccinees. This imbalance in infectivity of the DENV serotypes in the vaccine may be the cause of the lower efficacy of the vaccine to DENV1 and DENV2, and be an underlying cause of the increased risk of hospitalization seen in the year three follow-up in vaccinees 2–5 years of age, who were assumed to be seronegative at the time of vaccination [11].

Ultimately the results from the Dengvaxia clinical trials raise the question of whether Dengvaxia can effectively provide heterotypic protection against DENV and suggests that a more effective vaccine, that can generate heterotypic neutralizing antibodies against all four DENV serotypes, is still needed. Additionally, the target age group, children under the age of 9, could not receive Dengvaxia, leaving this vulnerable group unprotected against dengue.

## LATV TV003/TV005

The National Institute of Allergy and Infectious Diseases (NIAID) has been at the forefront of the development of a live-attenuated tetravalent vaccine (LATV) for over 15 years. Given that partial immunity to DENV can increase the risk of a more severe disease outcome with subsequent infection, multiple monovalent and tetravalent DENV vaccines were evaluated to identify candidates with optimal safety, infectivity and immunogenicity profiles with an overall goal to develop a LATV that could induce protection against all four DENV serotypes with a single dose. Through the implementation of recombinant DNA technology, two primary attenuation strategies were utilized to generate the vaccine viruses. The first strategy is the inclusion of deletions in the 3′ untranslated region (UTR) and the second strategy was the generation of structural gene chimerization (Fig. 1d). The prototype monovalent vaccine candidate, rDEN4Δ30 was generated through the introduction of a thirty nucleotide (Δ30) deletion in the 3′ UTR. rDEN4Δ30 contains all of the structural and non-structural proteins of a wild-type DENV-4 but it attenuated via the introduction of the 30-nucleotide deletion in the 3′ UTR (Fig. 1f) [19–22]. To generate the other DENV vaccines, a similar attenuation mechanism was utilized. For the DEN2 vaccine serotype (rDEN2/4Δ30) a chimerization strategy was utilized in which the prM and E genes of the DEN2 new guinea C virus were introduced into the rDEN4Δ30 vaccine candidate (Fig. 1e). A total of nine monovalent vaccine candidates were evaluated in flavivirus-naïve adult volunteers. Five different tetravalent admixtures were evaluated in clinical studies. The trials conducted on the monovalent viruses provided information that was critical for the choice of vaccine viruses regarding clinical, virological and immunological phenotypes of the viruses when administered singularly. This information served to inform the final development of the LATV vaccine. Important observations were made in these early studies, one such observation being that the chimerization strategy was highly attenuating for virus replication, while allowing for some viral replication necessary to induce an immune response. The rDEN2/4Δ30 candidate virus infected 100 % of subjects at a dose of 10^3^ p.f.u. when tested as a monovalent virus yet was the least infectious when combined into tetravalent formulations (rDEN1Δ30, rDEN2/4Δ30, rDEN3Δ30/3Δ31, rDEN4Δ30) [23, 24].

A balanced infectivity profile for all four components of the LATV is essential to ensure the induction of homotypic antibodies to each of the four DENV serotypes. To generate a more balanced infectivity profile two strategies were developed. The first strategy was to increase the dose of rDEN2/4Δ30 by tenfold [25]. This strategy is implemented in the TV005 formulation. TV003 and TV005 share the same four monovalent components and differ in the dosage of rDEN2/4Δ30 with TV003 being given at 10^3^ p.f.u. and TV005 being given at 10^4^ p.f.u. The second strategy was to increase the period for serological evaluation from 42 to 90 days post-vaccination. Increasing the dose of rDEN2/4Δ30 by tenfold was sufficient to compensate the higher HID_50_ of the vaccine component. A significantly higher percentage of TV005 recipients had detectable virus in blood post-vaccination than those that received TV003. The frequency of seroconversion improved from 76 % in those receiving TV003–97 % in those receiving TV005 [24]. In addition, the overall frequency of tetravalent antibody response following a single dose of vaccine increased from 74 % with TV003–90 % with TV005 [26].

In terms of antiviral responses after vaccination, sterilizing immunity can be defined as ‘inhibition or neutralization of subsequent infection by the virus against which one was vaccinated’ [27]. Although sterilizing immunity is not always necessary to achieve the end goals of reduced disease and morbidity, it can be a quantifiable clinical outcome and an indicator that the vaccination was successful at inducing a sufficient immune response. As previously described, Dengvaxia requires three doses and must be administered over a 12-month period. Viremia, particularly that of the DENV-4 component, was observed after the first dose and to lesser extents after the second and third doses. The second and third doses of Dengvaxia resulted in a boost in antibody litres to the DENV-1, DENV-2 and DENV-3 serotypes [28]. These data indicate that when administered to dengue-naïve individuals, Dengvaxia is not able to induce sterilizing immunity against later infection. To determine if breakthrough infection observed with Dengvaxia would occur with LATV, a second dose of TV003 and TV005 was administered 6 months after the first dose. After an initial dose of TV003, 75 % of vaccine recipients produced detectable levels of vaccine virus in the blood and 62 % of vaccine recipients developed a rash that is characteristic of dengue infection. No rash or viremia were observed after the second dose that was administered 6 months post-initial dose of TV003/TV005. After the second dose, only one recipient elicited detectable viral levels for 24 h. Following the second dose of vaccine a less than twofold increase in mean antibody titre to each of the four DENV serotypes was observed [27].

Neutralizing antibody responses and T-cell responses to TV003 and TV005 were measured 180 days after vaccination and were demonstrated to be sufficient to block boosting following subsequent vaccine administration as measured by the lack of further increase in antibody titres. To assess if the antibody levels would protect against natural DENV infection, a DENV-2 challenge model was developed in humans. The challenge strain rDEN2Δ30 was evaluated in ten healthy flavivirus-naïve subjects. To quantify the protective efficacy of TV003 against DENV-2 infection, subjects were challenged with rDEN2Δ30 6 months post-receipt of TV003 [29]. TV003 provided complete protection against viremia, neutropenia and rash induced by rDEN2Δ30 [29], and a DENV-3 challenge model is also being assessed and the results are in the process of being published. Additionally, Butantan Institute in Brazil in partnership with NIAID is carrying out phase-II and phase-III clinical studies with TV003 in dengue endemic countries. These results are quite encouraging regarding the potential for this LATV to protect against dengue in individuals already exposed to one or more DENV serotypes.

## TAK-003

TAK-003 (TDV) is a live-attenuated tetravalent dengue vaccine being produced by the Takeda pharmaceutical company and was originally designed and constructed by scientists at the US Division of Vector-Borne Diseases of the Centers for Disease Control and Prevention. The vaccine is based on an attenuated virus and chimeric viruses constructed using recombinant DNA technology. TAK-003 is based on a live-attenuated DENV-2 virus that provides the genetic backbone for all four of the vaccine viruses [30]. The first recombinant vaccine candidate was generated via the chimerization of the prM and E proteins of DENV-1 16 007 virus into DENV2 PDK-53 virus. Various vaccine constructs were generated by combining different wild-type viruses with PDK-attenuated vaccine viruses (Fig. 1h-j). Nine chimeric viruses were generated via the introduction of DENV-1 (16007), DENV3 (16562) or DENV-4 (1036) prM and E proteins with the DENV-2 (16681) and two genetic variants (PDK53-E and PDK53-V) [31]. DENV-2 PDK-53 was shown to replicate uniformly even when administered in combination and is speculated to induce a balance immunity against all four serotypes [32]. TAK-003 has a 0- and 3-month dosing schedule (Fig. 1k). Phase-I and II studies have demonstrated TAK-003 to be well tolerated and capable of inducing humoral responses against DENV serotypes 1–4, long-term antibody persistence and cross-reactive and multifunctional cellular responses [33]. Takeda Biologics TAK-003 vaccine has been granted priority review by the U.S. F.D.A. for the prevention of dengue disease caused by all four DENV serotypes in individuals 4 to 60 years of age and will be licensed in Indonesia in 2023 [7, 8]. Clinical trials conducted on the 0–3 month dosing schedule showed that two doses of TAK-003 showed an overall efficacy of 66.2 % (95 % CI, 49.1–77.5 %) in vaccine recipients who were seronegative prior to immunization [34, 35]. Efficacy levels against hospitalized dengue were 90.4 % and the efficacy against dengue haemorrhagic fever (DHF) was 85.9 % [35]. In an exploratory analysis, cumulative efficacy was assessed in three parts. Part one had a 12-month follow up after the second dose to assess the primary endpoint, part two had an additional 6 months to assess secondary endpoints and part three is an ongoing 3-year assessment of long-term efficacy and safety. Cumulative serotype specific efficacy was analysed for DENV-1 (69.0%), DENV-2 (90.8%), DENV-3(51.4%) and DENV-4, this latter one showing inconclusive results. Vaccine efficacy varied greatly by age group. In children 6–11 years of age, efficacy was 75.4 % while in children 4–5 years old age efficacy was 55.9 % [35]. In year 1, TAK-003 demonstrated an overall efficacy of 80.2 % (95 CI, 73.3–85.3 %) against Virologically Confirmed Dengue (VCD). In year 2, overall efficacy against VCD fell to 56.2 %. A total of 15 cases of severe dengue or DHF have been reported up to 2 years after the second dose and cumulative efficacy against DHF was 81.2 %. In the baseline seronegative group, analysis of geometric mean antibody litres from 9, 15 and 27 months demonstrated a trend of decreasing DENV-2 geometric mean litres over time (GMT) [35]. GMTs for DENV-1, -3 and -4 remained stable in vaccine recipients. Seropositivity rates were 91.3 % at 9 months and 85.9 % at 27 months. Overall, 27 months post-receipt of vaccination in children 4–16 years of age, TAK-003 prevented 72.7 % of symptomatic dengue cases 89.2 % of hospitalized dengue and 81.2 % of DHF [34]. The cumulative efficacy in vaccine recipients that were baseline seronegative was 67 and 74.8 % in seropositive individuals. Critically, the waning efficacy against DENV-2, which forms the backbone of TAK-003 cannot be overlooked and suggests that the TAK-003 may not fully replicate the protection provided by natural infection. In addition, data from year two demonstrated that there was higher efficacy in 6–11 year olds (60.6%) and 12–16 year olds (71.2) when compared to 4–5 year olds (24.5%) [33].

## TDEN F17/F19

The Walter Reed Army Institute of Research (WRAIR) in collaboration with GlaxoSmithKline (GSK) developed a live-attenuated, tetravalent dengue (TDEN) vaccine. The vaccine is composed of four DENV strains, DENV-1 (45AZ5), DENV-2 (S16803), DENV-3 (CH53489) and DENV-4 (341750) (Fig. 1l, m) and is administered in two doses given with a 6-month interval to flavivirus naïve adults (Fig. 1n). All four viruses were obtained from natural infections, isolated in C6/36 cells and attenuated through serial passages in PDK cells [36]. A total of 16 tetravalent formulations differing in their vaccine virus content, formulations 13 and 14 were selected for further evaluation. A new formulation, F17pre, was developed to optimize the neutralizing antibody response by using a higher primary dog kidney (PDK) cell passaged DENV-1 and a lower PDK cell passaged DENV-4 [37]. F17pre was designed as the precursor to the F17 formulation [38]. F13, F14 and F17pre underwent phase-II clinical trials. On day 10 after primary vaccination, 75, 31 and 31 % of subjects were viremic with formulations, respectively. Viremia was not detected in any subject following the second dose of the vaccine. Neutralizing antibody responses were measured for the formulations, 36, 40 and 63 % of vaccinated subjects developed tetravalent neutralizing antibodies after two doses of 13, 14 and F17, respectively. In a randomized phase-II clinical trial conducted in Puerto Rico, F17 elicited higher litres of neutralizing antibodies after a second dose and resulted in 100 % seroconversion to all serotypes in primed subjects [39]. To assess the safety and immunogenicity profile of the TDEN F17 vaccine, a pilot study was conducted in 6–7-year-old Thai children that were naïve for DENV 1–4 and JEV [36]. The results of the study showed that the vaccine is safe and immunogenic and as a result the study advanced to phase-I/II trial in infants aged 12–15 months. A total of 52.6 % of vaccine recipients seroconverted to the four DENV serotypes [40]. TDEN F17 has been shown to be safe and capable of eliciting an immune response in vaccine recipients 12 months to 50 years of age when administered in a two-dose regimen. TDEN F17 is currently undergoing a phase-I/II clinical study in children 6 to 9 years of age who were previously administered the vaccine. The 5-year follow-up will assess the immunogenicity of the two-dose regimen and that of a third dose administered 1 year after the second dose.

## DPIV

**Fig. 2.**
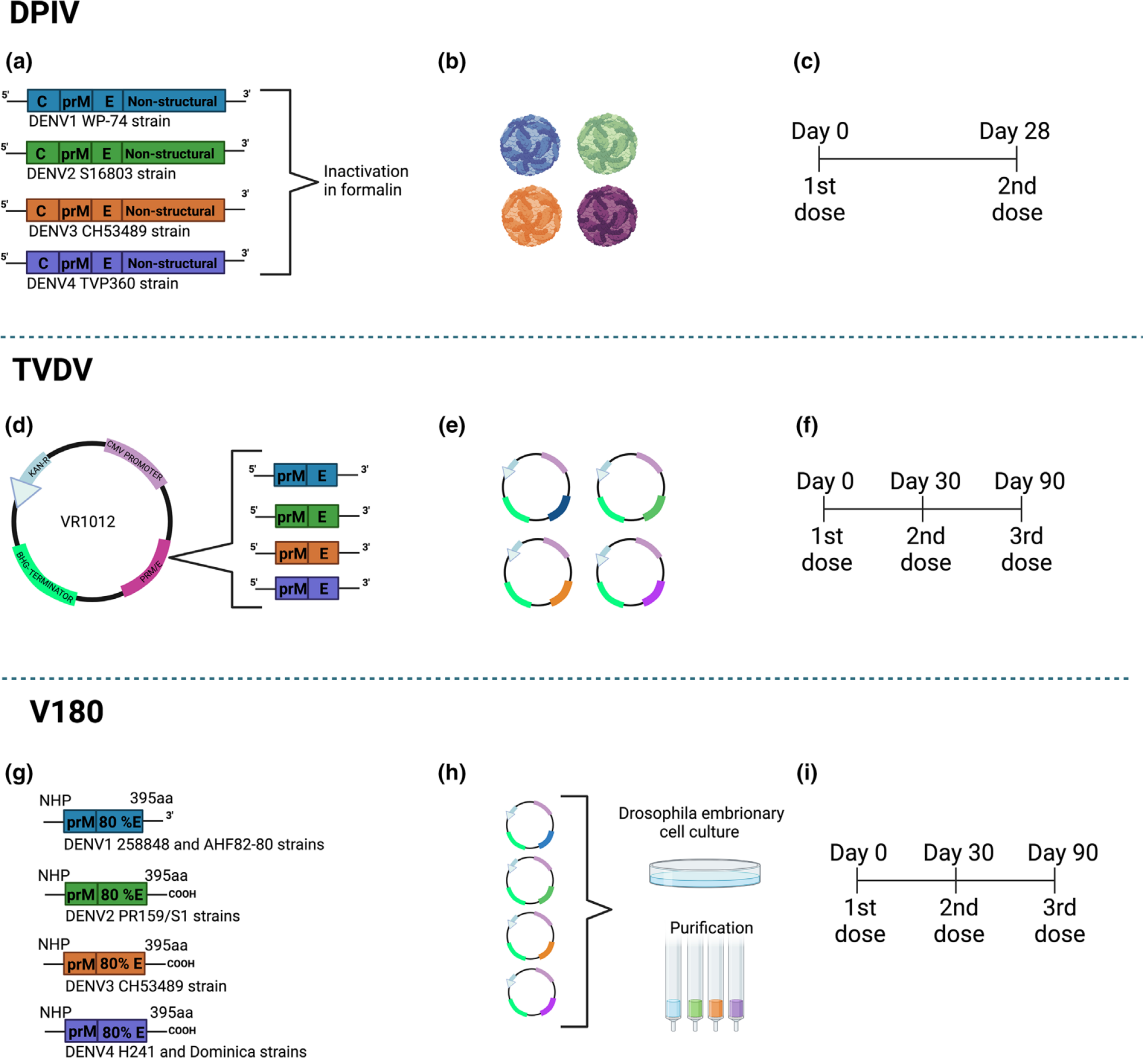
Development of live-attenuated vaccines by the Walter Reed Army Institute of Research (WRAIR), the U.S. Army Medical Research and Material Command and Merck and Co. Figure modified from Whitehead *et al*. 2007 [77] (**a**) Development of DPIV. Each DENV serotype was purified and formalin inactivated. (**b**) Representation of resulting virion production. (**c**) DPIV immunization regimen based on a two-dose schedule. (**d**) Development of TVDV vaccine. prM and E protein coding regions from serotypes representing DENV strains 1–4 are cloned into a VR1012 plasmid and co-administered with VAXFECTIN. (**e**) Schematic representing the resulting four DENV plasmid constructs. (**f**) Vaccine regimen for TVDV consisting of in a three-dose regimen beginning at day 0 followed by day 30 and ending at day 90. (**g**) Development Merck and Co., V180 vaccine. Genetic constructs of each DENV serotype were generated for viral sequences coding for the prM and 80 % of the E protein. (**h**) Resulting proteins were cloned into a pMttΔXho vector and cultured in Drosophila cell culture before undergoing purification. (**i**) Vaccine regimen for V180, which is administered in a three-dose regimen beginning with the first dose at day 0, the second dose at day 30 and the final dose at day 90. Diagrams were designed using Biorender.

The tetravalent dengue purified inactivated vaccine (DPIV) was developed by the Walter Reed Army Institute of Research (WRAIR) and is manufactured by the WRAIR Pilot Bioproduction Facility and is adjuvanted by GlaxoSmithKline (GSK) adjuvant systems. The vaccine is administered in two doses with an interval of 28 days (Fig. 2c) [41]. Initial vaccine development began with the DENV-2 S16803 strain, which was isolated from a patient and propagated in Vero cells. This purified inactivated virus (PIV) was safe when administered to mice, which developed 100 % seroconversion after the second dose [42]. PIV was subsequently tested on rhesus monkeys and was found to be safe and immunogenic. Subsequently, a tetravalent formulation based on inactivated viruses was developed using DENV-1 Westpac 74, DENV-2 S16803, DENV-3 CH53489 and TVP360 as the DENV-4 component (Fig. 1a, b). Viruses were propagated in Vero cells and underwent formalin inactivation and 0.01 % alum (aluminium hydroxide) as an adjuvant. The immunization schedule was composed of a dose of TPIV followed by a booster dose of a tetravalent live-attenuated vaccine (TLAV) [43]. The combination of TPIV/TLAV provided complete protection against DENV-3 challenge at 8 months post-vaccination. In a subsequent experiment, priming with TPIV elicited neutralizing antibodies against all four DENV serotypes. After challenge with each one of the four DENV serotypes, vaccinated animals exhibited no viremia [44]. Testing was conducted to find the best adjuvant for DPIV and vaccine developers used AS01_E_ (3-O-desacylcinomonophosphoryl lipid A) and AS03_B_ (oil-in-water) to overcome problems with immunogenicity caused by inactivation. In a phase-I clinical trial DPIV adjuvanted by AS01E/AS03B it was found that the vaccine elicited neutralizing antibody responses against all four DENV serotypes in dengue naïve adults, but this response decreased over time [45]. The humoral responses observed with the two-dose administration of the vaccine were robust, but it is unknown if DPIV is capable of providing long-lasting immunity against DENV. The inactivated nature of the vaccine may generate obstacles regarding immune responses to non-structural proteins [46, 47]. In a study conducted it was found that only envelope and capsid proteins were targeted by the immune system after immunization with DPIV. The vaccine was ultimately unable to control DENV infection in challenge assays carried out in rhesus macaques immunized with the vaccine. Importantly, vaccination with DPIV resulted in increased levels of viremia, AST, IL-10, IL-18 and in vaccinated monkeys [48]. This data indicates that vaccination may have triggered antibody-dependent enhancement of DENV infection.

## TVDV

The tetravalent DNA vaccine against dengue (TVDV) was developed by the U.S. Army Medical Research and Material Command [49]. TVDV is a DNA-based vaccine in which the prM and E protein coding sequences are clones in the VR1012 plasmid and are co-administered with VAXFECTIN as an adjuvant (Fig. 2d, e) in an immunization regimen of three doses (Fig. 2f) [50]. The DENV-1 strain was generated from the West Pacific 74 strain and when cloned into the VR1012 plasmid and administered to rhesus macaques was shown to protect the monkeys during challenge assays and was found to be immunogenic in phase-I clinical trial [51]. The DENV-2 antigen was generated by modification of the original DENV-2 construct by replacing the DENV-2 transmembrane and cytoplasmic sequences with those of the mouse lysosome-associated membrane protein. The DENV-3 antigen was derived from an Asian viral strain. Preclinical tests showed that the DENV3 vaccine strain elicited neutralizing antibody, moderate levels of specific IgG and conferred partial protection in challenge assays [50]. The genetic construction of DENV-4 was similar to that of DENV-2 yet there is a lack of reports on the preclinical tests of the DENV-4 component in its monovalent form. Vaxfectin was used an adjuvant to improve the immunogenicity of the vaccine. The vaccine constructs were prepared by combining equal amounts of the individual monovalent plasmids encoding the prM and E genes of DENV 1–4 and cloning them into the VR1012 plasmid. TVDV was tested for evaluation of safety and immunogenicity in white rabbits receiving two doses of the vaccine formulation. Animals were shown to seroconvert to all four DENV serotypes [52]. In the latest phase-I clinical trial conducted in 40 flavivirus naïve vaccine recipients, TVDV was shown to be safe and elicited IFN-γ anti-DENV T cell responses [51].

## V180

The V180 vaccine is a tetravalent vaccine based on the use of recombinant forms of DENV E and prM glycoproteins. The vaccine is currently in phase-I clinical trials and is being produced by Merck and Co. Genetic constructs of each of the DENV serotypes was generated via Reverse Transcriptase (RT)-PCR-based amplification of viral sequences coding for the prM and 80 % of the E protein (truncated E protein) that we cloned into a pMttΔXho vector (Fig. 2g, h) [53, 54]. The viral strains used were DENV1 258 848 and DENV1 Thailand AHF82-80, DENV2 strain PR159/S1, DENV3 strains CH53489 and D3H87 and DENV4 strains H241 and Dominica [54] and the vaccine is administered in a three-dose schedule (Fig. 2i).

In a phase-I, randomized, placebo-controlled, double-blind study that evaluated the safety, tolerability and immunogenicity of the vaccine it was found that the two V180 un-adjuvanted and the one aluminum-adjuvanted did not elicit a robust immune response while the six V180 formulations administered in combination with ISCOMATRIX elicited robust immunogenicity (GMT ≥150) [55]. It is currently unknown if the levels of antibody litres elicited in response to V180 would offer any clinical benefit given that the precise level of neutralizing antibodies needed for protection from DENV are not yet defined for vaccines.

## DSV4

**Fig. 3.**
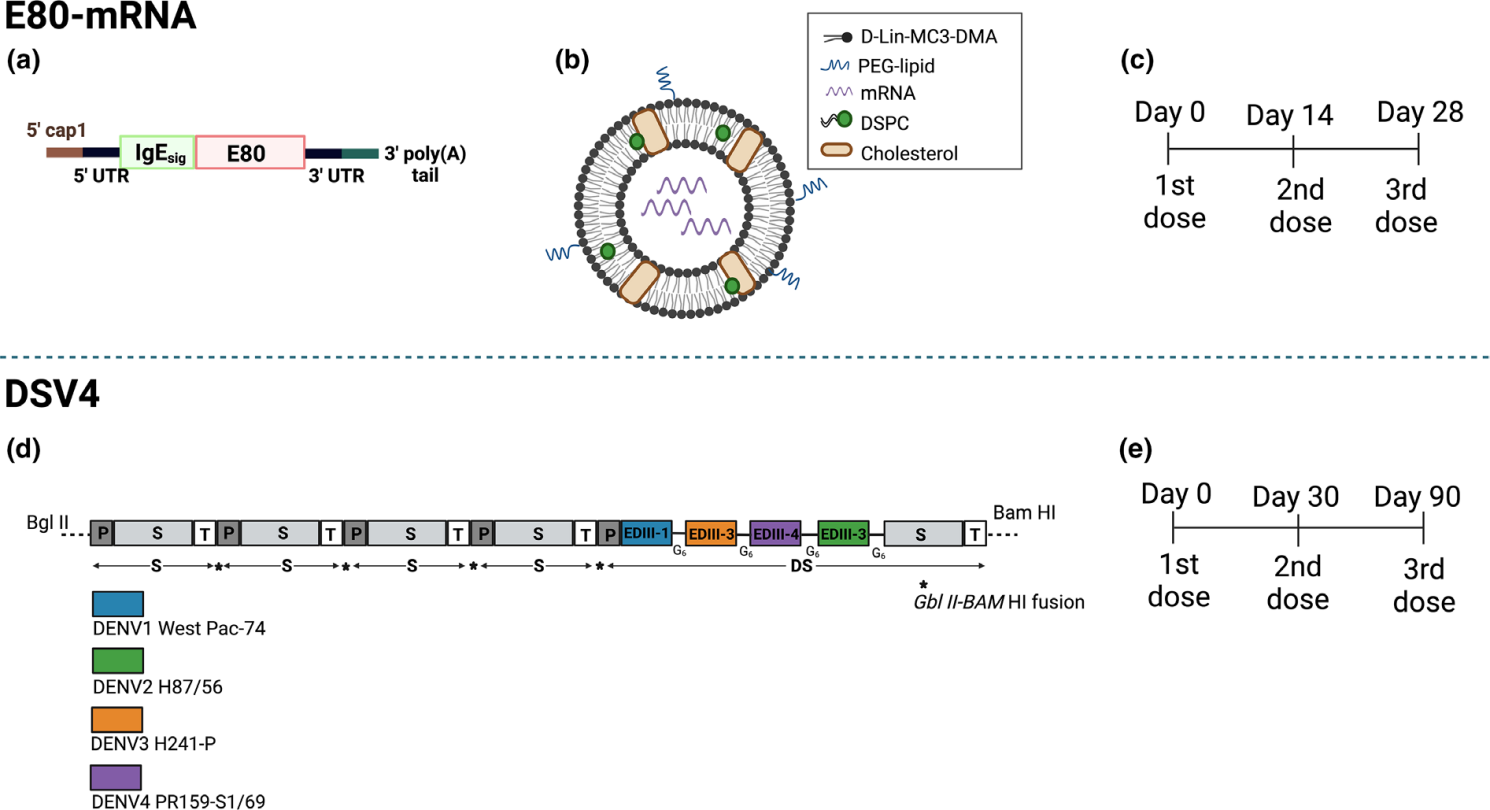
Development of E80-mRNA and DSV4 vaccines. (**a**) Development of E80-mRNA. RNA sequence containing a Cap1 (N7^m^GpppA^m^) sequence followed by a signal peptide sequence from human IgE followed by the E80 protein, a 3’ UTR and a 3’ polyA tail. (**b**) Representation of modified RNA packaged into a LNP. The LNP is composed of four lipids, d-Lin-MC3-DMA, DSPC, cholesterol and PEG-lipid. (**c**) Vaccination regimen for E80-mRNA. The vaccine is administered in a three-dose regimen with the first dose being administered on day 0, the second dose on day 14 and the final dose on day 28. (**d**) Development of DSV4 vaccine. DSV4 is designed using an in-frame fusion of DENV EDIII of each serotype and a hepatitis B surface (**s**) antigen co-expressed with unfused S antigen to form mosaic virus like particle s(VLPs) (**e**) Vaccination regimen for DSV4. The vaccine is administered in a three-dose regimen beginning with the first dose at day 0 followed by the second dose at day 30 and the third dose at day 90.

DSV4 is a tetravalent virus-like particle vaccine designed to display domain III of DENV envelope proteins. DSV4 was designed by using in-frame fusion of EDIII of all four DENV serotypes and hepatitis B surface (S) antigen and is co-expressed with unfused S antigen to form mosaic virus-like particles (VLPs) and is administered on a three-dose schedule (Fig. 3d, e). DSV4 induced levels of neutralizing antibodies against all four DENV serotypes in mice and conferred protection against a DENV4 challenge [56].

## E80 mRNA vaccine

The E80 mRNA vaccine is a modified mRNA-lipid nanoparticle (mRNA-LNP) vaccine developed by the Chinese Academy of Sciences. The vaccine was designed with a Cap1 (N7^m^GpppA^m^) sequence followed by a signal peptide sequence from human IgE followed by the E80 protein, a 3′ UTR and a 3′ polyA tail (Fig. 3a) [57]. The modified RNA containing the modified nucleoside 1-methylpseudouridine-5/-triphosphate was chemically synthesized and packed into LNPs (Fig. 3b). The LNP consisted of four lipids: d-Lin-MC3-DMA, DSPC, cholesterol and PEG-lipid combined at a molar ratio of 50 : 10 : 38.5 : 1.5. HEK293T cells were 52 % positive for E protein expression. The vaccine is administered on a three-dose schedule (Fig. 3c). E80-mRNA induced high levels of neutralizing antibody litres with an average PRNT_50_ of 13 000 in mice. The E80-mRNA conferred sterilizing immunity against a DENV-2 challenge in vaccinated immunocompetent mice and elicited reduced ADE activity in K562 cells infected with DENV-1, DENV-3 and DENV-4 [58].

## Challenges to DENV vaccine development

The development of an effective dengue vaccine is contingent on the ability of a vaccine to be effective against all four antigenically different DENV serotypes while eliciting equal levels of protection to all four. The inability of a vaccine to induce equal levels of neutralizing antibodies to all four serotypes can result in the induction of ADE. As a result, ADE presents a challenge in the development of a successful DENV vaccine. It has been reported that a specific range DENV antibody litres correlates with the risk of severe dengue disease but the major risk factor is previous infection with a heterologous DENV serotype in humans [59].

Current animal models for the study of dengue include non-human primates (NHP), immunocompromised mice and humanized mice. Of the animal models, the AG129 immunocompromised mice, which have somewhat capable antiviral immunity but have a dampened innate immune response, are the most commonly used to study the *in vivo* pathogenesis and response to challenge with DENV. A major challenge in the use of AG129 mice is their deficiency in interferon (IFN) alpha/beta and gamma receptor and lack of antiviral immune response [60]. Due to the inability to develop a full immune response, AG129 mice are not a suitable model for the validation of dengue vaccination efficacy and protection induced by the vaccine, while they have been quite useful to determine levels of viremia induced by the vaccine. Therefore there is a lack of an affordable animal that can recapitulate the immune responses to dengue virus.

Key questions remain unanswered with respect to how antibody responses are elicited during dengue vaccination. In addition to T-cell responses, the role of cell-mediated immunity (CMI) studies have been at the forefront of conversations in the dengue vaccine field. At the WHO-led consultation in 2008, it was recommended that exploratory studies be conducted in CMI [61]. However, it is challenging to apply a single universal standard for quantifying correlates of protection across all dengue vaccines. The fundamental differences between dengue vaccines result in difficulties to define appropriate correlates of protection for DENV infection. For example, inactivated vaccines may induce fundamentally different antibody responses when compared to live vaccines and even in individuals achieve the same neutralizing antibody (nAb) litres with the two different vaccines, the protective function and quality of the antibody responses may differ *in vivo* [62]. Another critical difference is that some of the DENV vaccines lack the DENV nonstructural genes or capsid proteins. This is of key importance due to CD8^+^ T cells primarily targeting nonstructural (NS) proteins [29, 63]. Live-attenuated vaccines without DENV NS proteins differ in the quality and nature of their T-cell responses. Markedly, live-attenuated vaccines that contain all DENV proteins have been shown to induce CD8^+^ T cells that elicit responses similar to the responses induced by natural infection [64]. Additionally, CD4^+^ T cells are capable of cytotoxic functions that may play a critical role in protection from severe disease [65].

## Final remarks

The health burden of dengue has increased dramatically on a global scale within recent decades. The need for a safe and effective dengue vaccine has never been more imperative. For this special issue of ‘10Q in virology’, we have made a thorough review of the state of the art in dengue vaccine development and emphasized the most important question that still remains unanswered in the dengue field: what is the contribution of each serotype to the severity of DENV infection and to vaccine efficacy? The ideal vaccine should provide long-term protection against all four serotypes, work effectively amongst all groups and be safe to administer to individuals regardless of serostatus.

Based on the available data from clinical trials, the NIAID LATV TV003/TV005 vaccine is the most promising vaccine in development. It provides robust levels of neutralizing antibodies in vaccinees, seroconversion to all four serotypes and is administered in a single dose, making it functional to administer in developing nations [66]. Given that there is not a single or universal correlate of protection to assess efficacy to DENV vaccines, the use of a challenge model becomes critical in evaluating the efficacy of a vaccine and we recommend that the use of a challenge model becomes the standard in evaluating correlates of protection to DENV vaccines. In addition to the use of challenge models, the NIAID LATV TV003/TV005 also investigated each individual vaccine component within the context of serotype to define immunogenicity [67].

Current research conducted by our group is evaluating the contribution of each vaccine serotype within the context of innate immunogenicity in primary human systems. Members of our team were able to identify features of innate immunity that define responses to the challenge virus [68]. Innate immune responses mediate the quality and quantity of T- and B-cell memory and therefore protective immune responses. DENV has been extensively characterized for its antagonism of innate immune responses [69, 70]. Interestingly, the role of innate immune responses has been historically understudied in DENV vaccine design. Within the context of innate immunity dendritic cells (DCs) play a key role in the sensing of viral pathogens and activating the adaptive immune response via stimulation of T-lymphocytes [71]. It is well known that during DENV infection, DCs become one of the main target cells for infection [72, 73]. The non-structural (NS) proteins of DENV have been characterized for their ability to antagonize innate immune responses in human cells including DCs via the targeting of type-I interferon responses through mechanisms such as the antagonism of the cGAS-STING pathway, amongst others [70, 73, 74]. Given that induction of an innate immune response is necessary to the induction of adaptive immunity, the role of DENV NS proteins that modulate the innate immune responses should be taken into consideration during vaccine design. In addition to DENV NS proteins targeting the innate immune system the NS proteins of DENV contain key epitopes that target CD8^+^ and CD4^+^ T cells. CD4^+^ T cells respond predominantly to the capsid and E proteins while CD8^+^ T cells respond predominantly to the NS3 and capsid [65, 75]. Both the structural and non-structural proteins induce T-cell mediated immune responses as the T-cell epitopes are distributed amongst structural and non-structural proteins. It has been described that during DENV vaccination, individuals who elicited high levels of the innate immune cytokine IP-10 upon initial vaccination demonstrated higher levels of protection against subsequent infection with DENV in a DENV challenge model [68].
